# Plant Defensins from a Structural Perspective

**DOI:** 10.3390/ijms21155307

**Published:** 2020-07-26

**Authors:** Valentina Kovaleva, Irina Bukhteeva, Oleg Y. Kit, Irina V. Nesmelova

**Affiliations:** 1 Laboratory of Molecular Genetic Markers in Plants, Ukrainian National Forestry University, 79057 Lviv, Ukraine; kovaleva@nltu.edu.ua; 2 Department of Physics and Optical Sciences and Center for Biomedical Engineering and Science, University of North Carolina at Charlotte, Charlotte, NC 28223, USA; ibukhtee@uncc.edu; 3 Interregional Academy of Personal Management, 79059 Lviv, Ukraine; oleg.kit.mail@gmail.com

**Keywords:** plant defensin, structure, dynamics, γ-core, NMR, x-ray, oligomer, dimer, antifungal, antibacterial

## Abstract

Plant defensins form a family of proteins with a broad spectrum of protective activities against fungi, bacteria, and insects. Furthermore, some plant defensins have revealed anticancer activity. In general, plant defensins are non-toxic to plant and mammalian cells, and interest in using them for biotechnological and medicinal purposes is growing. Recent studies provided significant insights into the mechanisms of action of plant defensins. In this review, we focus on structural and dynamics aspects and discuss structure-dynamics-function relations of plant defensins.

## 1. Introduction

Plants produce a variety of proteins and peptides as part of the innate defense against pathogens, including α/β-thionins, heveins, knottins, lipid transfer proteins, cyclotides, snakins, α-harpinins, glycine-rich peptides, and defensins [1]. Defensins are widespread in plants [2]. Plant defensins received their name because their structural and functional properties resemble those of insect and mammalian defensins [2]. They form a family of small cationic proteins (about 45–54 amino acid residues). Defensins are found in different parts of plants such as seeds, leaves, flowers, roots, and stems [3,4,5]. These proteins are constitutively expressed in the cell wall and extracellular space of seeds [4], xylem, stomata, and stomata cells in leaves [6] and the peripheral layers of roots [7], flowers [5], and fruits [8]. Such localizations indicate an important role for these proteins in the protection of the entry points of potential invaders [9]. Besides, the expression of defensins is induced by pathogen attack, wounding, and some abiotic stresses [8,10,11]. The most common activity of plant defensins is antifungal. However, the spectrum of plant defensin activities is quite broad, ranging from protection against bacteria, fungi, and viruses [12,13,14] to inhibition of protein synthesis [15], α-amylases [16], proteases [17], or ion channels [18].

Several classifications were proposed for the plant defensin family. Initially, plant defensins were divided into three groups based on their functional activity and morphogenic effects exerted on fungal hyphae [2]. Group I includes the “morphogenic” plant defensins that reduce hyphal elongation with an increase in hyphal branching [2,19,20], whereas the “non-morphogenic” plant defensins slow down hyphal extension without visible morphological distortions and form Group II. All other defensins that demonstrated weak or no antifungal activity were classified into Group III [16]. Later, several defensins were isolated from *Spinacia oleracea* leaves (So-D2 to So-D7) and were placed in Group IV [21]. Spinach defensins were active against Gram-positive and Gram-negative bacterial pathogens, as well as against fungi. The reduction of fungal growth occurred without hyphal branching [21]. The grouping of defensins into groups I-IV was based on two dozen sequences and was not suitable for the classification of the growing number of defensin sequences. Van der Weerden & Anderson carried out the phylogenetic analysis of 139 plant defensins and showed that they can be clustered into eighteen distinct groups [22]. While new defensins are still being actively discovered and the proposed grouping may change in the future, this analysis showed that defensins with similar activities often cluster together. However, the authors conclude that more data on defensin biological activity are needed to refine the grouping of defensins and use phylogenetic analysis to predict the activities of new and uncharacterized defensins. 

Additionally, plant defensins are divided into two classes based on the precursor structure [3]. Class I defensins are synthesized with an N-terminal signal sequence and a mature defensin domain. The signal peptide targets the defensin to the endoplasmic reticulum and then to the secretory pathway. Class I defensins provide the first line of defense against invading plant pathogens in the extracellular space. The less common class II defensins possess an additional C-terminal prodomain (CTTP) that targets the defensin to the vacuole of the plant cell and is removed subsequently [13,23]. The CTTP sequence of class II defensins is also important to prevent phytotoxicity effects of defensin against other host cells [23]. Analogous negative prodomains are located at the N-terminus of insect and vertebrate α- and β-defensins [24]. Structurally, mature proteins of class I and class II defensins have a similar three-dimensional fold as monomers.

Plant defensins have a common fold as monomers, which is known as the cysteine-stabilized αβ (CSαβ) fold. Other proteins that demonstrate the CSαβ fold include insect defensins [25,26], scorpion toxins [27,28], and the sweet-tasting protein brazzein [29]. Given that plant defensins are small proteins with similar, compact, and stable structures supported by at least four disulfide bonds, the broad range of activities that they demonstrate is striking. Recent studies provided significant insights into the mechanisms of action of plant defensins. In this review, we highlight the structural and dynamics features of plant defensins in relation to their function.

## 2. Primary Sequence

**Figure 1:**
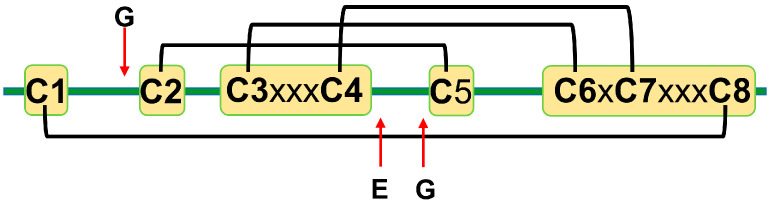
A schematic representation of plant defensins. Letters denote conserved amino acids. The conserved number of amino acids (x—any amino acid) separates cysteines C3 and C4 (three amino acids), C6 and C7 (one amino acid), and C7 and C8 (three amino acids). Letters and red arrows denote conserved amino acids and their approximate locations in the amino acid sequence, respectively. Black brackets represent disulfide bonds.

The amino acid sequence identity between different plant defensins varies from less than 35% to more than 90% [12,22]. Most plant defensins have eight conserved cysteine residues (C1 to C8). The number of amino acid residues between cysteines C1 to C3 and C4 to C6 varies from three to more than ten, whereas the number of amino acid residues between other cysteines is conserved to three, one, and three: C3-x-x-x-C4, C6-x-C7, and C7-x-x-x-C8, where x denotes any amino acid [22]. Disulfide bonds between cysteine residues follow the following pattern: C1-C8, C2-C5, C3-C6, and C4-C7 [14] (Figure 1). A few exceptions to the eight-cysteine paradigm exist, e.g., defensins PhD1 and PhD2 from the flowers of *Petunia hybrida* possess ten cysteine residues that form five disulfide bonds [5,30]. The first additional cysteine residue is located between cysteines C1 and C2, whereas the second additional cysteine residue is located between cysteines C2 and C3. The additional disulfide bond in PhD1 does not change the cysteine connectivity pattern or tertiary structure as compared to other plant defensins.

**Figure 2:**
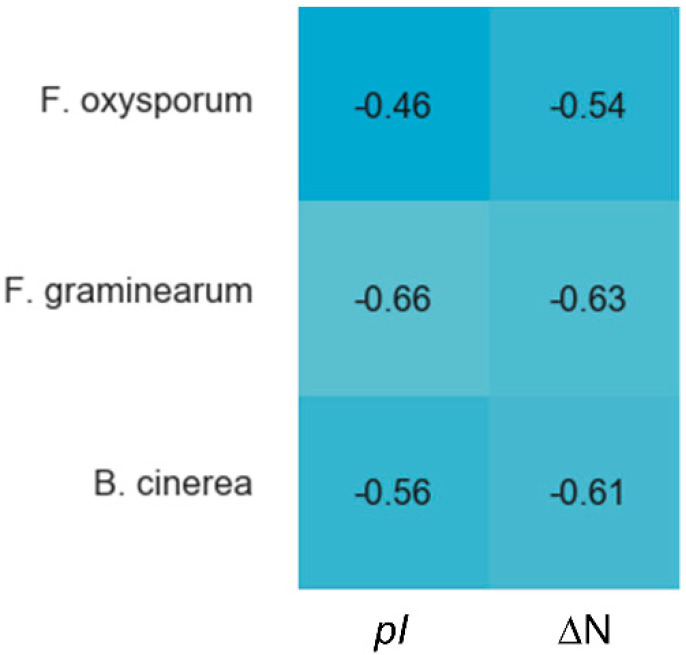
Correlations between the overall charge or pI and antifungal activity of plant defensins. The heat map shows Pearson coefficients calculated in Spyder using a built-in Pandas correlation function for *F. oxysporum, F. graminearum,* and *B. cinerea.* The overall charge is represented as the difference between positively (Arg, Lys) and negatively (Asp, Glu) charged residues, ΔN. The blue color indicates a negative correlation, e.g., a defensin active at smaller concentrations (IC50). Lighter shades of blue correspond to stronger correlations. Coefficients from −0.50 to −0.70 are classified as a moderate correlation, yet the trend is present.

Cysteines are the only fully conserved residues in plant defensins. In addition to cysteines, other residues frequently occurring in plant defensins include two glycines and glutamic acid, as schematically indicated in Figure 1 with arrows, and, often, a charged, typically basic residue (arginine or lysine) at the N-terminus and an aromatic residue (phenylalanine or tryptophan). Such a low number of conserved amino acid residues suggests that other factors, e.g., the relative content of charged versus hydrophobic residues and/or their distribution in the sequence, may have functional importance. In particular, plant defensins are cationic proteins. We examined whether the correlation between the antifungal activity of plant defensins and their overall charge and isoelectric point pI was present. The overall charge was represented by the difference between positively (Arg, Lys) and negatively (Glu, Asp) charged amino acids, ΔN. We considered three types of fungi, *Fusarium oxysporum*, *F. graminearum*, and *Botrytis cinerea*, because only for these fungi the activities measured in relatively similar experimental conditions were reported for more than ten different plant defensins. Table 1 lists the activities used in our analysis. Pearson correlation coefficients presented in the heat map shown in Figure 2 indicate that the antifungal activity is stronger (smaller inhibitory concentrations IC50) when the overall positive charge or the value of pI is larger (negative correlation), albeit the correlation is moderate because the value of Pearson coefficient is in the range between −0.5 and −0.7.

## 3. Secondary and Tertiary Structures

The Protein Data Bank (PDB) contains three-dimensional structures of 24 plant defensins determined by NMR spectroscopy or x-ray crystallography (Table 2). Despite the significant variability of amino acid sequences, experimental structures of plant defensins display high similarity as monomers, suggesting that this fold is important for their biological activity. All structures, except for pea defensin Psd2 [50], can be superimposed within less than 3Å for Cα atoms of secondary structure motifs, with the majority of structures displaying less than 2Å deviation. 

**Figure 3:**
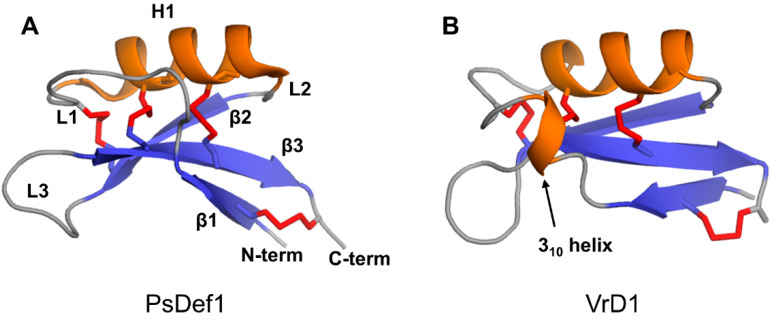
Three-dimensional structures of two plant defensin monomers. Structures of (**A**) PsDef1 (PDB code 5NCE) [51] and (**B**) VrD1 (PCB code 1TI5) [52] are shown in cartoon representations. Disulfide bonds are shown using red sticks. Letters indicate secondary structure elements on the structure of PsDef1 as follows: loops L1-L3, beta-strands β1-β3, alpha-helix H1. All plant defensins adopt a common disulfide-supported CSαβ fold.

The defensin topology consists of one alpha-helix H1 and three antiparallel beta-strands (β1, β2, and β3) arranged in the order β1-H1-β2-β3 as shown in Figure 3A on the example of pine defensin PsDef1 (PDB code 5NCE) [51]. The secondary structure elements are connected by loops L1, L2, and L3, where loops L1 and L3 are the longest. In VrD1 defensin from the mung bean, a 3_10_ helix is formed in the middle of loop L1 (Figure 3B). The formation of the 3_10_ helix in VrD1 may result from the replacement of the highly conserved glutamic acid E26 by arginine. This substitution disrupts conserved hydrogen bonds between strand β1 and helix H1 observed in other defensins [52]. In defensins Rs-AFP1 and Rs-AFP2, loop L3 contains a type VI β-turn [53,54,55]. Both the 3_10_ helix in VrD1 and the type VI β-turn in Rs-AFP1 and Rs-AFP2 seem to have no functional implications and are unique to these defensins.

Four conserved disulfide bonds link secondary structure elements assembled into a compact globular three-dimensional structure. Disulfide bond C1-C8 connects the N-terminus to the C-terminus. Disulfide bond C2-C5 connects loop L3 to beta-strand β2. Disulfide bonds C3-C6 and C4-C7 tether helix H1 to beta-strand β3. Due to the insertion of exactly one amino acid between cysteines C6 and C7 located on beta-strand β3 (Figure 1), the two disulfide bonds C3-C6 and C4-C7 orient in the same direction towards the alpha helix. This parallel arrangement of C3-C6 and C4-C7 disulfide bonds in plant defensins is referred to as cis arrangement [74,75]. On the contrary, in trans-defensins, the two cysteines located on the beta-strand are vicinal, and, therefore, the disulfide bonds point in the opposite direction from beta-strand β3, linking it to different secondary structure elements [74,75].

Disulfide bonds are known to increase the thermodynamic stability of globular proteins [76]. The presence of four disulfide bonds may explain the high structural stability of defensins at extreme temperatures and pH values [14,77,78]. Indeed, FTIR (Fourier Transform InfraRed) spectroscopic data demonstrated that the content of α-helical and beta-sheet secondary structures in PsDef1 was preserved up to 80 °C, and no thermally induced unfolding was detected. Upon cooling from 80 °C back to 25 °C, the FTIR spectrum was similar to the one observed at 25 °C before heating, showing the reversible behavior of PsDef1 [10]. Furthermore, PgD5 defensin from *P. glauca* retained 71% of antifungal activity against *Verticillium dahlia* after 30 min of treatment at 75 °C and 61% at 100 °C [44], whereas rice defensin OsAFP1 demonstrated a negligible loss of activity after heating at 100 °C for 10 minutes [79], and defensins Rs-AFP1 and Rs-AFP2 were unaffected by exposure to 100 °C for 15 min [80]. Defensin-like antifungal peptide NRBAP from *Phaseolus vulgaris* is reported to retain activity following the exposure to 100 °C for 30 min and to extreme pH values, ranging from 0–1 (obtained by dissolving the NRBAP in hydrochloric acid) to 12 [81]. An antifungal peptide from brown kidney beans with a molecular weight of 5.4 kDa and N-terminal sequence highly homologous to plant defensins retained full antifungal activity from 20 °C to 80 °C and from pH 3 to 12 [82]. A defensin-like antifungal peptide from French beans retained antifungal activity after heating to 90 °C for 20 min and in the pH range of 4–10 [83], and the antifungal activity of Cp-thionin II from *Vigna unguiculata* remained unaffected by heat treatment at 100 °C for 20 min, demonstrating excellent thermal resistance [84].

Thermal and pH stability data highlight the high tolerance of plant defensins to changes in environmental conditions and suggest that retaining their structural scaffold is important for functional activity. Indeed, chemical reduction and alkylation of *N. occidentalis* defensin 173 (NoD173) resulted in the loss of ability to permeabilize human tumor cells (U937 cells), suggesting a critical role for its three-dimensional structure, at least for this activity [70]. 

## 4. The Oligomerization of Plant Defensins and Cell Membrane Targeting

To date, the best-studied mechanism of plant defensin action is related to cell membrane disruption, which frequently involves two linked processes, the binding of defensin to phospholipids driven by the overall positive charge of defensin and the oligomerization of defensin [65,66,67,68,69,72]. The disruption of dimer formation by site-directed mutagenesis of key residues greatly reduced the capacity of NaD1 defensin to kill the filamentous fungus *F. oxysporum* with an approximately 5-fold increase of its IC50 value [65]. The R39A mutant of NaD1 defensin lost the ability to oligomerize in the presence of phosphatidic acid (PA) and showed an attenuated ability to inhibit the growth of *Candida albicans* [67]. Similarly, the NaD1 R40E mutant demonstrated reduced binding to the 4-phosphate moiety of phosphatidylinositol 4,5-bisphosphate (PIP_2_) and decreased oligomerization that correlated with substantially reduced antifungal activity [66].

Strikingly, most NMR structures show that defensins are monomers in solution, whereas the majority of x-ray crystallographic structures show that they form dimers and higher-order oligomers (Table 2). One can argue that solution NMR structures of defensins are solved at acidic pH conditions that promote the monomeric state for these cationic proteins. Indeed, plant defensins with known three-dimensional structures have theoretical pI values in the range of 7.7 to 9.8, with the majority of them having pI greater than 8, and an extreme case of ZmD32 defensin from Z. mays with the pI value of 11. At the same time, crystal packing forces can somewhat favor a particular sub-state of a protein, e.g., oligomerization. Nonetheless, growing biochemical and biophysical experimental evidence shows that plant defensins have the propensity to oligomerize.

The presence of dimers and higher-order oligomers was detected by SDS-PAGE analysis and/or chemical cross-linking experiments for plant defensins Rs-AFP1 and Rs-AFP2 [20], NaD1 [65], EcgDf1 from *Erythrina crista-galli* [85], SPE10 [86], J1-1 from *Capsicum genus* [87], OsDEF7 and OsDEF8 from *O. sativa* [88], TPP3 [72], MtDef5 from *M. truncatula* [89], and rice defensin OsAFP1 [73]. Furthermore, the dimers of NaD1 defensin in solution were detected using small-angle x-ray scattering analysis and analytical ultracentrifugation [65], whereas the presence of PsDef1 dimers was detected by NMR diffusion measurements [10]. These data suggest that the oligomerization of plant defensins is their intrinsic property. 

**Figure 4:**
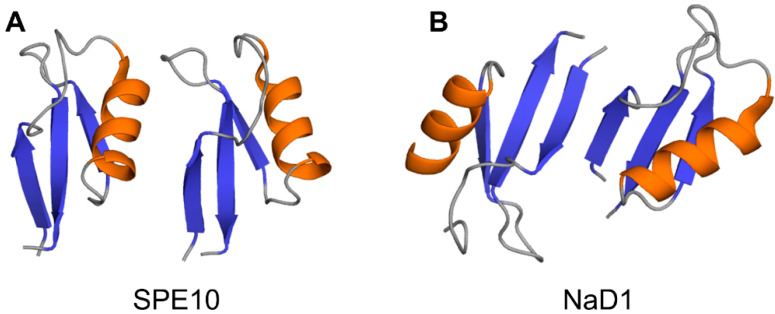
Three-dimensional structures of plant defensin dimers. Cartoons present two different monomer arrangements in the dimer structures of plant defensins (**A**) SPE10 (PDB code 3PSM) [71] and (**B**) NaD1 (PDB code 4ABO) [65]. In SPE10 dimer, the two monomers are packed against each other approximately in a side by side manner, with a beta-sheet of one monomer facing the alpha-helix of another monomer. In NaD1 dimer, the β1 strands of the two monomers interact, forming an extended beta-sheet structure comprising three beta-strands of each monomer.

Crystallographic structures show that the building block of plant defensin oligomers is a dimer [66,67,68,69,70]. The number of experimental structures of defensin dimers is limited; however, it is apparent that defensins may adopt different dimer conformations (Figure 4). In the case of SPE10 defensin, the two monomers are packed against each other approximately in a side by side manner [71] (Figure 4A). Thus far, such an arrangement of monomers is reported only for SPE10 defensin. The dimerization of SPE10 defensin is electrostatically driven by the favorable arrangement of positively charged residues R36 and R40, located on strands β2 and β3 in one monomer, and negatively charged residues D21 and D22, located on the alpha-helix in another monomer. Furthermore, spatially, residues R36 and R40 form a conformational triplet motif with tryptophan W42, where the side-chain of R36 is held by the side-chain of W42 through cation-π interaction. Crystallographic structures of several other defensins, including NaD1, TPP3, OsAFP1, NoD173 [70] and NsD7 [68,69], demonstrate a more symmetrical dimer with an extended β-sheet stabilized by hydrogen bonds formed between the residues from beta-strands β1 in one monomer and residues located on the same beta-strand in another monomer [65,66,67,68,69,70,72,73] (Figure 4B). Such geometry of a dimer seems to facilitate the formation of higher-order oligomers because all crystallographic structures show that the higher-order oligomers are formed of β1-strand interface dimers [66,67,68,69,70].

**Figure 5:**
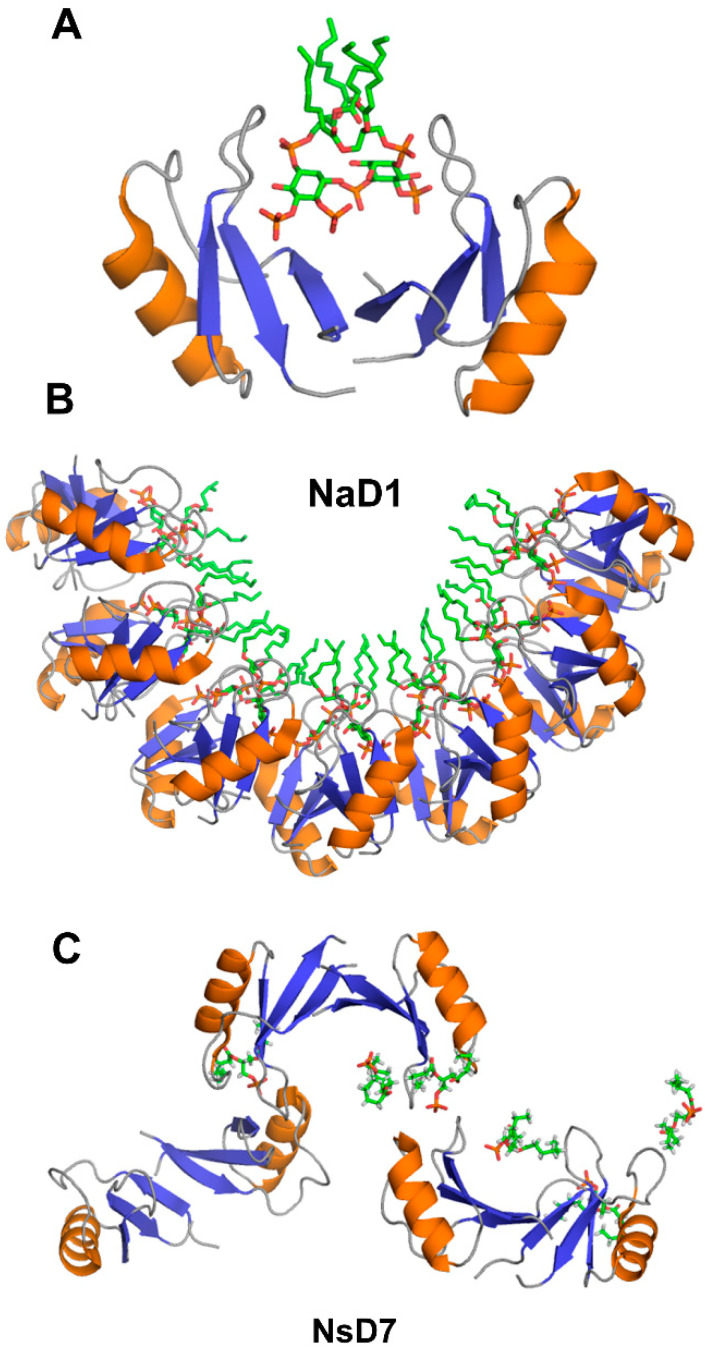
Three-dimensional structures of plant defensins in complex with phospholipids. (**A**) NaD1 defensin dimer forms a “catioinic grip” to bind phosphatidylinositol 4,5-bisphosphate (PIP_2_) (PDB code 4ABO) [65]. (**B**) Seven dimers of NaD1 defensin form a horseshoe-like oligomeric structure upon binding to PIP_2_ (PDB code 4ABO) [65]. (**C**) NsD7 defensin forms an extended helical defensin–lipid oligomer upon binding to phosphatidic acid (PDB code 5KK4) [68].

The target cell membrane disruptive activity of defensins relies on the recognition of anionic moieties, such as glycoproteins, sphingolipids, or phospholipids [13,90]. The structural insight into this process comes from x-ray crystallographic studies of defensin-phospholipid oligomers [65,66,68,69,70,72]. The dimerization of NaD1 through β1 beta-strands leads to the formation of a large continuous positively charged surface that is instrumental for accommodating negatively charged head groups of phospholipids [65,66,67]. In the structure of NaD1 with PIP_2_, two PIP_2_ head groups fit simultaneously in the positively charged groove of NaD1 dimer, in which the orientation of two monomers creates a so-called “cationic grip” holding onto PIP_2_ head groups (Figure 5A). Likewise, the dimers of defensins TPP3, NsD7, NoD173, and OsAFP1 adopt a cationic grip conformation [68,69,70,72,73]. 

The oligomerization of NaD1 is also primarily electrostatically driven. The NaD1 oligomer is formed as a horseshoe-like assembly of seven NaD1 dimers cooperatively binding 14 PIP_2_ molecules (Figure 5B). Defensin NsD7, possessing a 91.5% amino acid sequence identity to NaD1, also forms a horseshoe-like oligomeric structure with PIP_2_, albeit containing six dimers and 12 PIP_2_ molecules [69]. The overall topology of the defensin-phospholipid complex depends both on the properties of defensin and phospholipid. Apparently, the NaD1-type dimer (β1-strand interface) has more flexibility for the relative monomer orientation within the dimer, aiding the various modes of binding different phospholipids. In contrast to PIP_2_, both NaD1 and NsD7 form an extended helical defensin–lipid oligomer with phosphatidic acid [68] (Figure 5C). NsD7 oligomer demonstrates two binding sites for PA molecules. In addition to the cationic grip, which is denoted as the type II binding site, the type I PA-binding site is located at the connecting point between two neighboring defensin dimers with the PA molecule bridging the two defensin monomers from adjacent dimers. Unlike PA, larger PIP_2_ molecules containing three phosphate groups cannot bind to the type I site, which leads to a different arrangement of dimers in NaD1-PIP_2_ oligomer. Furthermore, unlike NaD1–PIP_2_ and NsD7–PA complexes that can only extend in one direction, the NaD1–PA complex displays the ability to extend in two directions, forming a carpet-like structure [67]. In contrast, as revealed by TEM (transmission electron microscopy), TPP3 defensin forms long, string-like fibrils with diameters of approximately 10 nm only in the presence of PIP_2_ [72]. These fibrils further laterally associate by stacking against each other horizontally, resulting in “bundles” of fibrils.

The formation of extended defensin-phospholipid structures by NaD1 and several other defensins discussed above suggests that they may use a “carpet” model of cell membrane disruption. According to the “carpet” model [91], peptides first bind to negatively charged moieties on the target membrane and cover it in a “carpet”-like manner. In the next step, peptide monomers align on the membrane surface so that their hydrophilic surfaces face the phospholipid head groups or water molecules, and the hydrophobic residues orient toward the hydrophobic core of the membrane without inserting into it. Finally, after reaching a threshold concentration, peptides permeate/disintegrate the membrane by disrupting the bilayer curvature. Note that before the collapse of the membrane, the formation of holes in the membrane and the passage of low molecular weight molecules through these holes can occur. However, in contrast to the barrel-stave model, these holes are transient.

The main property of peptides that utilize the carpet mechanism is the net positive charge spread along the peptide chain, preventing their assembly with the hydrophilic peptide surfaces facing each other to form a pore-like structure. In this regard, cationic plant defensins possess the required attribute for the carpet mechanism of action. Further supporting the “carpet” model of action, plant defensins are active at micromolar concentrations (Table 1), which would be a requirement of the “carpet” mechanism [91]. Furthermore, peptides that form transmembrane pores are typically alpha-helical, whereas plant defensins have a mixed α/β secondary structure (Figure 3). For example, molecular dynamics (MD) simulations of the interaction of defensin PsDef1 with model POPC/POPG bilayers show that PsDef1 attaches to the membrane surface by interacting electrostatically with lipid polar heads, without deep penetration into the hydrophobic tail zone [92]. A positively charged PsDef1 defensin induces changes in the lipid distribution along the membrane, resulting in the formation of zones with different electrostatic potentials that can cause deformation or distortion of the membrane corresponding to the “carpet” model. Although first introduced to describe the mechanism of antimicrobial peptides [91], the carpet model can be extended to antifungal peptides as fungal cell walls are decorated with negatively charged glycoproteins and lipids [93,94,95]. Interestingly, antimicrobial peptides have no preferred secondary or tertiary structure, show the arbitrary length, and can be either linear or cyclic upon binding to the membrane, as long as a certain level of hydrophobicity and number of positive charges is present. The compact, disulfide-stabilized structure of plant defensin monomers is suitable to serve as a building block of extended carpet-like assemblies.

Despite the presented above compelling evidence, it is not yet clear whether the “carpet” mechanism of action is common for all plant defensins. For example, several plant defensins, including Rs-AFP2, α-PT from *Triticum aestivum*, Hs-AFP1, and Dm-AMP1, demonstrated a biphasic activity on the target membrane. These defensins were able to cause strong and cation-sensitive membrane permeabilization in Neurospora crassa at relatively high protein concentrations ranging from 10 to 40 µM, indicative of the “carpet” mechanism of action. However, at lower protein concentrations in the 0.1 to 1 μM range, a weaker and more cation-resistant membrane permeabilization was observed [96]. The latter suggested a binding-site mediated membrane permeabilization followed by the insertion of the defensin into the membrane as the altered membrane’s permeability to Ca^2+^ and K^+^ ions and organic molecules like SYTOX Green was detected [96]. Moreover, the SPE10 defensin monomeric mutant D38N showed a similar capacity of antifungal activity to the wild-type protein. However, different from the wild type, D38N induced morphological changes in the inhibited fungi hyphen, indicating a distinct antifungal mechanism [71]. Given that the SPE10 dimer is uniquely formed (Figure 4A), it is possible that plant defensins may adopt different antifungal mechanisms under different oligomerization states [71].

Collectively, the information on defensin oligomerization as well as their interactions with membrane lipids is still limited. New structures of plant defensins with different phospholipids, accompanied by functional studies, are required to build a comprehensive picture of how the plant defensins target the pathogen cell membrane.

## 5. Structural Motifs of Plant Defensins Important for Antifungal Activity 

### 5.1. γ-core Motif

**Figure 6:**
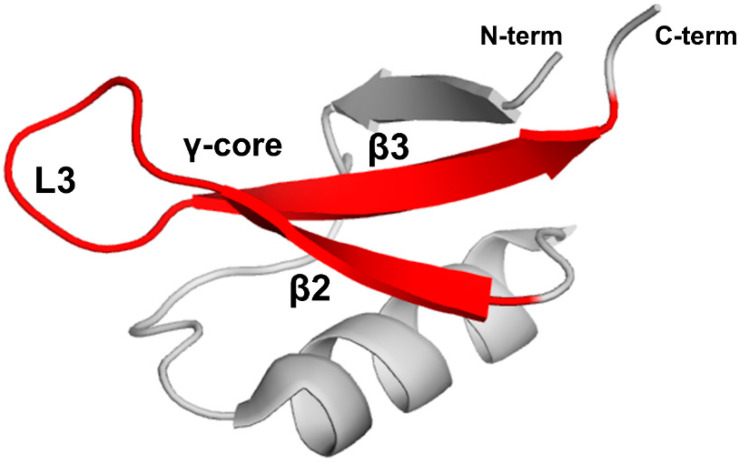
The γ-core motif. Introduced based on the consensus between all disulfide-containing antimicrobial peptides [97,98], the γ-core motif comprises two full-length beta-strands, β2 and β3, and loop L3 connecting them as shown by the red color on the three-dimensional structure of PsDef1 defensin (PDB code 5NCE) [51].

Stereospecific multiple sequence alignment (MSA) revealed a sequence motif, termed the γ-core motif, which is common to all disulfide-containing antimicrobial peptide classes from organisms separated by profound evolutionary distances [97,98]. The γ-core motif consists of two antiparallel beta-strands and a loop region connecting them. Additionally, the specific characteristics of the γ-core motif include: (1) a length of 8-18 amino acid residues; (2) conserved GXC or CXG amino acid residue triads within the sequence isoforms; (3) net cationic charge; (4) a periodic placement of charged and hydrophobic amino acid residues leading to amphipathic geometry; (5) participation in one to four disulfide bonds. This motif may constitute the entire peptide or only part of the protein [97]. Plant defensins possess the γ-core motif (X1-3GXCX3-9C…). Although some authors include only the GXCX3-9C sequence in the γ-core motif (hence, selecting shorter versions of γ-core motif for functional studies in some cases), based on the consensus between all disulfide-containing antimicrobial peptides [97,98], the γ-core motif includes full-length β2 and β3 strands and the loop L3 between them (Figure 6).

Several structure-activity studies indicate that the major determinants of the antifungal and antibacterial activity of plant defensins reside in the γ-core motif [42,54,79,89,99]. Furthermore, the γ-core motif peptides derived from defensins RsAFP-2, MtDef4, MtDef5, MsDef1, So-D2, Vu-Def from *Vigna unguiculata*, BcDef from *Brugmansia x candida*, PνD1 from *P. vulgaris*, and the tomato defensin SolyC07g007760 demonstrated antifungal and antibacterial activity at micromolar concentrations [21,42,99,100,101,102,103,104]. Additionally, specific regions within γ-core motifs of MsDef1 and MtDef4 defensins contribute to their different modes of antifungal action [105].

The MSA analysis suggested that the γ-core motif developed as a result of its critical functions in mediating protein-membrane interactions in the course of host defense against pathogens [98]. Consistent with this idea, the “cationic grip”, identified as a phospholipid-binding motif in crystallographic structures of several defensins [65,66,67,68,69,70,72,73] discussed above, is formed by amino acid residues located within the γ-core motif. 

The γ-core motif of plant defensins may confer the direct membrane-activity and/or serve as a structural scaffold, to which complementary determinants of functional activity are added. For example, the γ-core motif of MtDef1 alone is not sufficient for antifungal activity, whereas the full protein is [42]. The replacement of the γ-core motif of MsDef1 with that of MtDef4 substantially increased the antifungal activity of MsDef1 [42]. A structure-function analysis using 24-mer peptides, spanning the entire amino acid sequence of defensin HsAFP1 shows that the γ-core and its adjacent regions are important for inhibitory activity towards fungal biofilms [63]. 

### 5.2. Loop L3 of the γ-core Motif

The main physicochemical features responsible for the functional importance of the γ-core motif include the net positive charge and the periodic placement of charged and hydrophobic amino acid residues [42]. Increasing the positive charge of the PνD1 defensin’s γ-core motif derived peptide by replacing two aspartic acids D37 and D38 with two arginines significantly enhanced its antifungal activity [99]. Many charged residues are located in the L3 loop of the γ-core motif connecting beta-strands β2 and β3. The L3 loop of the γ-core motif of MetDef4, containing amino acid residues R-G-F-R-R-R, four of which are positively charged arginines forming a patch of the positively charged surface, interacts with phosphatidic acid [61]. Mutagenesis experiments provided strong evidence that the R-G-F-R-R-R loop is the translocation signal mediating the entry of MtDef4 into fungal cells. The RGFRRR loop is conserved in a large majority of plant defensins. The substitution of the native sequence of NaD1 with R-G-F-R-R-R switched the lipid-binding preference of the resulting defensin from PIP_2_ to PA [106]. The replacement of positively charged arginine R44 in the L3 loop of RsAFP1 defensin by a neutral glutamine amino acid reduced the antifungal potency, while substitution of the neutral valine V39 by arginine increased the antifungal potency [54]. While increasing the net positive charge seems to be a universal rule for increasing the activity of defensin, the specificity for the fungus target should be taken into account when molecular physicochemical properties are fine-tuned. The V39 replacement by arginine made Rs-AFP2 defensin more active against the fungi *F. culmorum*, *Nectria haematococca*, and *V. dahlia*, but less active against *Phoma betae* [54].

The L3 loop of SPE10 defensin is an important determinant of its oligomerization [71]. Additionally, SPE10 mutant with amino acid residue substitution D38N in loop L3 showed full activity towards *Bipolaris maydis* with an IC50 comparable to that of wild type protein. However, in contrast to wild type SPE10, the reduced hyphal elongation was accompanied by highly increased hyphal branching. Likewise, positively charged arginine R37 and H36 located in the L3 loop are critical for the oligomerization of MtDef5. Mutating these residues to alanine completely abolished the ability of MtDef5 to induce membrane permeabilization and inhibit fungal growth [89,107]. 

### 5.3. Loops L1 and L2

In addition to the γ-core motif, some studies indicated the importance of residues located in loop L1 for the functional activity of plant defensins. This is not surprising, because loops L1 and L3 are near each other and connected by the disulfide bond C2-C5. In the case of RsAFP2 defensin, T10G and G9R substitutions of amino acid residues in loop L1 showed noticeable functional effects [54]. The T10G mutant showed reduced activity on *F. culmorum*, whereas the G9R mutant showed no significantly increased activity on *F. culmorum* in the low ionic strength medium and a 2-fold increase of activity synthetic in the high ionic strength medium (synthetic medium fungi with added salts). In addition to the L3 loop of the γ-core motif, loop L1 of Psd1 defensin is involved in the binding of mimetic systems of the *F. solani* membrane [108]. In particular, residues F15 and T16 located at the end of loop L1 are responsible for the specific interaction of Psd1 with glucosylceramide. Interestingly, in the case of sugar cane defensin Sd5, NMR chemical shift changes resulting from the interaction with model membranes cluster mainly in loop L1, and, additionally, in the part of helix H1, in the C-terminal region, and loop L2 [60], forming two distinct membrane-binding sites. First, an unspecific binding site comprises residues from loop L1, helix H1, and the C-terminus that demonstrate changes both in the presence of phosphatidylcholine:glucosylceramide (PC:CMH) and dodecylphosphocholine (DPC). Second, a specific binding site comprises residues from loop L2 that only change in the presence of glucosylceramide. The involvement of the C-terminal region and loop L2 in lipid binding is not common to all plant defensins and may result from specific structural and dynamic properties of Sd5 defensin [60]. 

## 6. Structural Features of Plant Defensins Important for α-Amylase Activity 

The insecticidal properties of plant defensins closely correlate with their ability to inhibit insect digestive enzymes, such as α-amylase and proteases, which play a crucial role in the breakdown of plant starch and proteins, respectively. Over a thousand defensins are isolated from different taxonomic groups of plants; however, not all of them demonstrate α-amylase inhibition [19]. The α-amylase inhibitory activity is described for plant defensins VrD1 [52,109], PsDef1 [51,110], VuD1 from *V. unguiculata* [16], SIa1, SIa2 and SIa3 from *Sorghum bicolor* [111], γ1-H thionin [56,112], and TvD1 from *Tephrosia villosa* [38]. These defensins, added at micromolar concentrations, inhibit the activity of insect amylases; however, they are ineffective against α-amylases of mammals, plants, bacteria, and fungi. Only γ1-Н thionin is known to inhibit α-amylase from human saliva [112]. The following discussion is based on computational modeling and docking studies of defensins TvD1, VrD1 and γ1-H thionin and α-amylase from *Tenebrio molitor* (mealworm beetle) larvae (TMA) [38,52], VuD1 defensin and α-amylase from *Zabrotes subfasciatus* (ZSA) [16], and PsDef1 defensin and α-amylase TMA that we carried out for this review.

**Figure 7:**
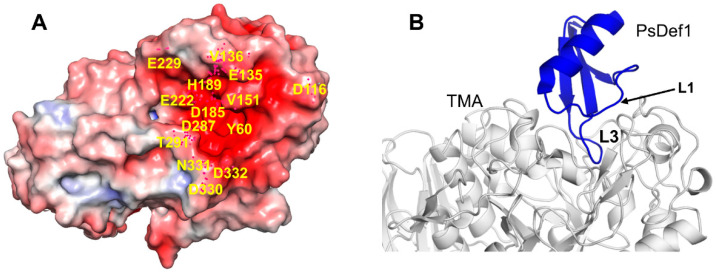
Docked structure of plant defensin PsDef1 and TMA. (**A**) Electrostatic potential is shown on the surface of α-amylase from *Tenebrio molitor* (mealworm beetle) larvae (TMA) (PDB code 1JAE) [113]. The blue color represents a positive charge, and the red color represents a negative charge. The active site of TMA is highly negatively charged. Residues within and surrounding the catalytic site discussed in the text are labeled on the structure. (**B**) The computationally docked structural model of PsDef1 shows that loop L3 enters the active site of TMA, and loop L1 forms additional contacts supporting the PsDef1-TMA complex. The NMR structure of PsDef1 (PDB code 5NCE) was docked into the X-ray structure of TMA using the ClusPro web server [119]. Protein interactions were evaluated using the PDBsum web server [120].

The best structurally characterized insect α-amylase is the TMA α-amylase [113]. It has three domains: A (residues 1–97 and 160–379), B (residues 98–159), and С (residues 380-417). The active center of TMA is located in the cleft at the interface between domains A and B. It includes three catalytic residues D185, E222, and D287, a calcium-binding residue H189 and several other conserved residues involved in substrate recognition and orientation. Residues located in the active center of TMA and its vicinity create a surface of the strong negative potential that attracts positively charged molecules, such as plant defensins (Figure 7A). 

Computational docking models of plant defensins VrD1, γ1-H thionin, and alpha-TvD1 show that loop L3 (of the γ-core motif) inserts into the active site of TMA, thereby preventing the substrate from reaching the catalytic site [38,52]. The shape complementarities between TMA and VrD1 or γ1-H are superior to those generally observed for TMA–inhibitor complexes, and VrD1 or γ1-H thionin form 20 or 18 hydrogen bonds with TMA, respectively [52]. All three defensins form hydrogen bonds with the catalytic residues of TMA: VrD1 and γ1-H-thionin with E222 and D287, and alpha-TvD1 with D185 and D287 as shown for other TMA protein inhibitors [114,115,116,117]. The comparison of defensins Vrd1 and γ1-H thionin to defensins Rs-AFP1 and Ah-AMP1, which do not inhibit α-amylase, shows that the length of loop L3, defining its conformation compatible with TMA binding, as well as the concentration of positively charged residues in loop L3, are critical for binding [52]. The mutagenesis study of defensin VrD2 that has no activity against α-amylases further confirms the importance of the length and amino acid residue composition of loop L3 for the inhibition of α-amylase. The VrD2 chimera, in which the five-residue motif G-M-T-R-T of defensin VrD1 replaced the equivalently located four-residue motif D-D-F-R in loop L3 of defensin VrD2, containing two negatively charged residues, acquired the activity against TMA [59]. The replacement of the same four-residue motif in TvD1 defensin with the five-residue motif from VrD1 also resulted in enhanced TMA α-amylase inhibitory activity as in defensin VrD2 [38]. Interestingly, in contrast to VrD2 that does not possess any insect α-amylase inhibitory effect, the wild type TvD1 has the same amino acid residues D-D-F-R in loop L3 and exhibits α-amylase inhibitory activity. Thus, other amino acids outside loop L3 also play a role in α-amylase inhibition. In alpha-TvD1, several amino acid residues from loop L1, including T16, T17, and G12, form hydrogen bonds with TMA, while F15 forms the π–π stacking interactions with the side-chain of W57 of the TMA, strengthening the binding [38].

Previously, we reported that PsDef1 defensin inhibits the activity of α-amylase from the larvae of pine pest *Panolis flammea* [51]. Here, we used the crystallographic structure of TMA (PDB code 1JAE) [118] to examine the structural basis of PsDef1 binding to α-amylase. The docked structure of PsDef1 in the complex with TMA shows that loop L3 fully occupies the active site of TMA and forms hydrogen bonds and ionic and non-bonded contacts with the residues of the catalytic center of TMA (Figure 7B). The catalytic residue D287 of TMA forms a hydrogen bond with G40 and the π-π stacking interactions with F36 of PsDef1. In the catalytic pocket, H37 of PsDef1 forms hydrogen bonds with K188 and E229 of TMA, and the salt bridge formed by H37 and E229 residues further strengthens the interaction. Residues R41 and K42 located at the end of loop L3 form several hydrogen bonds with TMA residues T291, N331, D332, and E135. Also, several residues from loop L1, including K11, G12, Y13, and N16 further stabilize the PsDef1-TMA complex by forming hydrogen bonds with residues E135, V151, Y60, and D330 of TMA, respectively. While the relative orientation of PsDef1 defensin and TMA somewhat differs from the docked structures of VrD1 and γ1-H thionin, the common feature of these complexes is the leading role of loop L3 in defensin-TMA interaction.

The docking analysis of defensin VuD1 and α-amylase ZSA that shows a 61% sequence identity with TMA revealed that K1 from VuD1 interacts with residue D204 inside the catalytic site of ZSA. In silico mutation of K1 to glycine stops complex formation between ZSA and VuD1. In the case of VuD1, the interaction of loop L3 does not occur at the catalytic site, but somewhere on the surface [16].

Given the limited number of structural models derived computationally and the lack of experimental structures of defensin-α-amylase complexes, it is premature to conclude a unifying structural theme in plant defensin binding to α-amylases. The analysis of the available structural models of plant defensins with mealworm beetle α-amylase TMA suggests that loop L3 enters the active site of α-amylase and loop L1 further supports the defensin-α-amylase interaction. Defensin VuD1 shows a different mode of binding to the ZSA α-amylase. While loop L3 still forms contacts with ZSA, the interaction of N-terminal lysine with one of the catalytic residues of ZSA is primarily responsible for enzyme inhibition. Generally, both the overall positive charge and the structural complementarity play an important role in the α-amylase inhibitory activity. Indeed, several studies of other protein α-amylase inhibitors established that steric factors, surface charge distribution, and chelating properties of the inhibitor contributed to its specificity [114,115,116,117].

## 7. Intracellular Targets of Plant Defensins

The intracellular targets of plant defensins are not well studied. However, a few examples, e.g., defensins NaD1, MtDef4, HsAFP1, MtDef5, PνD1, and Psd1, show that, in addition to targeting the cell membrane, plant defensins can also internalize into the intracellular environment, suggesting that they may act on intracellular targets [22,42,107,121,122,123]. Reported examples demonstrate a general theme: positively charged surfaces of plant defensins cause the initial, electrostatically driven binding to different moieties of opposite charge, whether on the membrane or intracellularly. Confocal microscopy revealed nucleic acid complexes with defensin MtDef5. MtDef5 defensin variant with the H36A, R37A, H93A, and R94A amino acid substitutions in its γ-core motif exhibits markedly reduced DNA-binding ability [107]. Interestingly, it is shown that the antibacterial effect of ostrich β-defensins (that are related to plant defensins) is associated with their ability to transit the cytoplasmic membrane, access the cytoplasm, and interact with bacterial DNA [124]. PsDef1 defensin bound phosphotyrosine (pTyr) in the pull-down experiments using pTyr affinity matrix and pine seedlings extracts [125]. Additionally, it was shown that both endogenous and recombinant PsDef1 specifically interact with pTyr-Sepharose. While PsDef1 defensin does not contain known pTyr-binding domains, it is highly positively charged at neutral рН, which may explain its high affinity to pTyr. It is possible that phosphorylated sites in proteins can be targets for defensins. While similar physicochemical properties of plant defensins suggest that they may have common intracellular targets as well, more experimental studies are needed to verify this idea.

## 8. Dynamics Properties of Plant Defensins

The role of protein dynamics in the functional activity of plant defensins is suggested by the fact that many functionally important residues are located in the loops [60,126]. Currently, the information on the dynamics of plant defensins comes mostly from NMR relaxation studies, which report that both the picosecond to the nanosecond and the microsecond to millisecond motions are present in plant defensins [47,48,50,51,60,108,126]. Note that in proteins the microsecond to millisecond motions are typically marked as the most functionally relevant [127,128,129,130,131]. It is not surprising that in such small, disulfide-stabilized, compact molecules as plant defensins, overall, the microsecond to millisecond conformational dynamics is observed in similar regions, though the degree of mobility varies between different defensins [47,48,50,51,60,108,126]. In this regard, the defensin molecule can be visualized as consisting of two parts. A more dynamic part is formed by the two longest loops, L1 and L3, and nearby regions, including the end of beta-strand β2 and the beginning of the beta-strand β3 (e.g., part of the γ-core), and the N-terminal part of the helix H1. A less-dynamic part comprises the rest of the molecule [47,48,51,60,108]. Naturally, due to the disulfide bond C2-C5 connecting loops L1 and L3, these loops show correlated motions [108]. Furthermore, despite the presence of the disulfide bond, the second conserved cysteine in all plant defensins displays a significant conformational exchange, and the cysteines forming the bond C2-C5 are frequently unobservable or broadened in comparison to other signals in NMR spectra [47,51,60,108]. In Lc-def defensin, despite systematic experimental condition optimization, NMR signals of the residues C14-C20 in loop L1 remained broadened beyond detection, indicative of significant conformational rearrangements on the timescale of the NMR experiment [47].

The observation of increased dynamics in the part of defensin molecule formed by loops L1 and L3 and nearby regions is in agreement with the results that show the involvement of this region in binding events, such as binding lipids, α-amylases, or oligomerization [38,52,59,65,66,67,68,69,70,71,72,73,89,93]. The direct evidence of the correlation between lipid binding and protein dynamics is provided for Psd1 defensin [108]. In the presence of model membranes, the regions of Psd1, directly involved in the interaction with a membrane (in particular, amino acid residues C14, F15, H36), showed reduced conformational exchange, likely due to the stabilization of a specific membrane-bound conformation of Psd1 [108]. Furthermore, in the agreement with the functional relevance of loops L1 and L3, the normal mode analysis shows that the dimerization of PsDef1 diminishes the mobility of loop L1 residues in slow modes but leaves the high-frequency motions in loop L1 and the mobility of loop L3 minimally perturbed [10].

The degree of mobility differs between different defensins, in part resulting from the difference in the length of the loops. For example, loop L1 in AtPDFL2.1 defensin is only four amino acid residues long. NMR relaxation measurements show that the structure of AtPDFL2.1 defensin is essentially rigid, with merely loop L3 and the second conserved cysteine (C14) located in loop L1 possessing marked flexibility [48]. Defensin Psd1, whose L1 loop has seven amino acid residues, displays a significant conformational exchange in loops L1 and L3 and the β2 strand [108,126]. In addition to loops L1 and L3, defensin PsDef1 shows significant dynamics for residues located in the N-terminal half of helix H1, at the end of strand β2 leading to loop L3, and at the beginning of strand β3. The contribution of conformational exchange to NMR relaxation rates in PsDef1 is significant, with values above the median observed for about 28% of all residues [51]. Finally, NMR relaxation data show that defensin Sd5 is highly dynamic on the millisecond timescale in several regions, with at least two distinct regimes with exchange rate constants of 490 and 1800 reciprocal seconds [60].

Studies at different temperatures reveal that some plant defensins may sample a large conformational space within the cysteine-stabilized α/β fold. In PsDef1, about 25% of all residues populate discrete alternative conformations that are less than 5 kcal/mole apart in terms of the free energy of the state. These conformations originate from different protonation states of the side chain of histidine H37, causing the formation or disruption of the hydrogen bond between side chains of H37 and Y44 residues, as well as the reorientation of H37 that affects neighboring residues [51]. Such conformations could be present in other defensins with highly conserved histidine at the position equivalent to H37 in PsDef1.

An extreme case of large-scale conformational rearrangements is presented by Sd5 defensin [50,60]. Unlike a typical well-folded globular protein, the Sd5 structure does not have an extensive hydrophobic core and is stabilized by contacts among surface-exposed polar and hydrophobic side chains. Increasing temperature changes the fine balance between these contacts, and Sd5 undergoes a millisecond time-scale interconversion between its native, low energy state, and an alternative excited conformational state that has less favorable enthalpy and yet is more compact than the native state. The observed dynamics and NMR chemical shift changes show that in the course of this interconversion the Sd5 α-helix undergoes a significant rearrangement, possibly transforming into an extended β-structure. Such a major rearrangement suggests that the Sd5 defensin belongs to a class of metamorphic proteins that can exist in two well-ordered yet structurally distinct conformational states [50]. The closest example of a metamorphic protein that has a disulfide-stabilized compact structure is a chemokine lymphotactin (XCL1) that alternates between α/β fold and fully beta-sheet conformation [132,133,134]. Interestingly, lymphotactin variants restricted to the conserved α/β fold chemokine fold lose their antimicrobial activity, suggesting that metamorphic folding of XCL1 is required for antimicrobial activity [134]. Moreover, the metamorphic form of XCL1 also permeates fungal model membranes and exhibits anti-*Candida* activity in vitro [132].

Although dynamics studies of plant defensins are sparse, dynamics properties may contribute to the function and specificity of plant defensins. Studies reveal that Psd1 and Sd5 defensins interact with membranes via the mechanism of conformational selection [60,108,126]; hence, the dynamics govern the efficiency of membrane recognition and disruption. Dynamics is expected to play roles in other activities of plant defensin as well, and further studies are needed to reveal its contributions to different defensin functions. 

## Figures and Tables

**Table 1:** Inhibitory concentrations for plant defensins against *F. oxysporum*, *F. graminearum*, and *B. cinerea*.

Defensin and Source	pI	ΔN	IC50 (µM)			Experimental Conditions	Reference
			Fusarium Oxysporum	Fusarium Graminearum	Botrytis Cinerea		
Rs-AFP1 *Raphanus sativus*	8.3	+3	2.6 2.6–5.2		1.4 1.4 1.4	½ PDB, T = 24 °C SM, T = 28 °C SM, T = 28 °C	[31] [4] [20]
Rs-AFP2 *Raphanus sativus*	8.7	+6	0.4 0.4	0.2–0.5 0.4	0.4 1.7 0.4 0.4	½ PDB, T = 24 °C ½ PDB, T = 24 °C SM, T = 28 °C SM, T = 28 °C PDB, T = 24 °C SM, T = 24 °C	[31] [19] [4] [20] [18] [32]
Ah-AMP1 *Aesculus hippocastanum*	7.6	+1			4.3	½ PDB, T = 24 °C	[19]
Dm-AMP1 *Dahlia merckii*	7.6	+1			2.2 14	½ PDB, T = 24 °C PDB, T = 22 °C	[19] [33]
Ct-AMP1 *Clitoria ternatea*	8.1	+3			3.5	½ PDB, T = 24 °C	[19]
Hs-AFP1 *Heuchera sanguinea*	8.2	+3			1.0	½ PDB, T = 24 °C	[19]
Psd1 *Pisum sativum*	7.6	+1	>19			PDB, T = 25 °C	[34]
Psd2 *Pisum sativum*	8.1	+3	>19			PDB, T= 25 °C	[34]
PhD1 *Petunia hybrida*	8.5	+6	0.4		1.4	½ PDB, T = 24 °C	[5]
PhD2 *Petunia hybrida*	8.3	+5	1.4		1.9	½ PDB, T = 24 °C	[5]
VrD1 *Vigna radiata*	8.7	+6	1.0–3.4			T = 28 °C	[35]
SPE10 *Pachyrrhizus erosus*	7.5	+1	>18		>18	PDB, T = 28 °C	[36]
VaD1 *Azuki Bean*	8.9	+7	5.8–10			not listed	[37]
TvD1 *Tephrosia villosa*	7.8	+2	1.2			PDB, T = 28 °C	[38]
PDC1 *Zea mays*	8.1	+3		~1.1		PDB, T = 28 °C	[39]
NmDef02 *Nicotiana megalosiphon*	8.1	+3	1			PDB, T = 28 °C	[40]
PsDef1 *Pinus sylvestris*	8.9	+7	0.5–0.7		~0.1	PDB, T = 23 °C	[41]
MsDef1 *Medicago sativa*	8.1	+3		1.2–2.3 2-4 1.5–3.0		PDB, T = 24 °C SM SM, T = 24 °C	[18] [42] [32]
MtDef2 *Medicago truncatula*	6.8	-1		>19 6-9		PDB, T = 24 °C SM, T = 24 °C	[18] [32]
MtDef4 *Medicago truncatula*	8.5	+6		0.75–1 0.75–1.0		SM SM, T = 24 °C	[42] [32]
Sm-AMP D1 Sm-AMP D2 *Stellaria media*	7.0 7.5	+1 +1	0.4	0.5	1.0	T = 22 °C	[43]
PgD5 *Picea glauca*	8.5	+5	1.9		0.7	PDB, T = 22 °C	[44]
PpDFN1 *Prunus persica*					2.9	1% glucose in H_2_O, T = 20 °C	[45]
NaD1 *Nicotiana alata*	8.7	+6	0.4 1	1 ± 0.5 0.5	1	PDB, T = 22 °C ½ PDB, T = 25 °C ½ PDB, T = 25 °C	[5] [11] [46]
NaD2 *Nicotiana alata*	8.5	+5	5	2			[46]
Lc-def *Lens culinaris*	7.8	+2			9.25	½ PGB, T = 25 °C	[47]
AtPDFL2.1 *Arabidopsis thaliana*	7.5	+1		4		¼ PDB, T = 25 °C	[48]
ZmD32 *Zea mays*	11	+10		1 ± 0.7		½ PDB, T = 25 °C	[11]
OefDef1.1 *Olea europaea*	9.1	+8	0.4 ± 0.1	1.6 ± 0.6	0.7 ± 0.3	PDB, T = 24 °C	[49]

PDB—potato dextrose broth, PGB—potato glucose broth, SM—synthetic fungal medium.

**Table 2:** Defensins with known three-dimensional structures and their respective PDB codes and experimental conditions.

Defensin and Source	PDB Code	Oligomeric State	Method	Experimental Conditions	Reference
γ1-P thionin *Triticum aestivum*	1GPS	Monomer	NMR	H_2_O/D_2_O pH = 4.0, T = 22, 32 °C 1–1.5 mM	[56]
γ1-H thionin *Hordeum vulgare*	1GPT	Monomer	NMR	H_2_O/D_2_O pH = 4.0, T = 22, 32 °C 1–1.5 mM	[56]
Rs-AFP1 *Raphanus sativus*	1AYJ	Monomer	NMR	H_2_O/D_2_O pH = 4.2, T = 32.3 °C 1.3 mM	[55]
Rs-AFP2 *Raphanus sativus*	2N2R	Monomer	NMR	H_2_O/D_2_O pH = 4.0, T = 15−35 °C 2 mg/mL (~0.3 mM)	[53]
Ah-AMP1 *Aesculus hippocastanum*	1BK8	Monomer	NMR	H_2_O/D_2_O pH = 4.1, T = 19.8 °C 3.1 mM	[57]
Psd1 *Pisum sativum*	1JKZ	Monomer	NMR	10 mM sodium phosphate pH = 4.0, T = 17, 27, 37 °C 1.8 mM	[58]
Psd2 *Pisum sativum*	6NOM	Monomer	NMR	20 mM phosphate 10 mM NaCl pH = 5.0, T = 25 °C 1 mM	[50]
PhD1 *Petunia hybrida*	1N4N	Monomer	NMR	H_2_O/D_2_O pH = 3.1, T = 7, 37 °C 0.94 mM	[30]
VrD1 *Vigna radiata*	1TI5	Monomer	NMR	50 mM phosphate pH = 6.0 T = 10, 15, 20, 25 °C 2 mM	[52]
VrD2 *Vigna radiata*	2GL1	Monomer	NMR	50 mM phosphate pH = 6.0 T = 15, 20, 25 °C 2 mM	[59]
Sd5 *Saccharum officinarum*	2KSK	Monomer	NMR	5 mM sodium phosphate pH = 4.0 T = 25 °C 0.2–1.0 mM	[60]
MtDef4 *Medicago truncatula*	2LR3	Monomer	NMR	20 mM Tris pH 4.4 T = 20 °C 1 mM	[61]
Lc-def *Lens culinaris*	2LJ7	Monomer	NMR	H_2_O/D_2_O pH = 5.0, T = 27, 55 °C 0.5–1.0 mM	[47]
AhPDF1.1 *Arabidopsis halleri*	2M8B	Monomer	NMR	H_2_O/D_2_O pH = 4.5, T = 25, 35 °C 1.3 mM	[62]
AtPDFL2.1 *Arabidopsis thaliana*	2MZ0	Monomer	NMR	H_2_O/D_2_O pH = 6.5, T = 25 °C 0.2 mM	[48]
HsAFP1 *Heuchera sanguinea*	2N2Q	Monomer	NMR	H_2_O/D_2_O pH = 4.0, T = 25 °C 2 mg/mL (~3 mM)	[63]
PsDef1 *Pinus sylvestris*	5NCE	Monomer	NMR	10 mM acetate pH 4.5, T = 33 °C 0.4-0.7 mM	[51]
ZmD32 *Zea mays*	6DMZ	Monomer	NMR	H_2_O/D_2_O pH = 3.5, T = 25 °C 1 mM	[11]
NaD1 *Nicotiana alata*	1MR4 4AAZ 4AB0 4CQK 6B55	Monomer Dimer 7-mer of dimers	NMR x-ray x-ray x-ray x-ray	H_2_O/D_2_O T = 25, 30, 37 °C 1.5 mM complex with PIP_2_complex with PA	[64] [65] [65] [66] [67]
NsD7 *Nicotiana suaveolens*	5KK4 5VYP	Dimer 6-mer of dimers	x-ray	complex with PA complex with PIP_2_	[68] [69]
NoD173 *Nicotiana occidentalis*	6MRY	6-mer of dimers	x-ray		[70]
SPE10 from *Pachyrhizus erosus*	3PSM	Dimer	x-ray		[71]
TPP3 *Solanum lycopersicum*	4UJ0	Dimer	x-ray		[72]
OsAFP1 *Oryza sativa*	6LCQ	Dimer	x-ray		[73]
